# Latent effects of fibronectin, *α*5*β*1 integrin, *α*V*β*5 integrin and the cytoskeleton regulate pancreatic carcinoma cell IL-8 secretion

**DOI:** 10.1038/sj.bjc.6602132

**Published:** 2004-08-31

**Authors:** A G Lowrie, D M Salter, J A Ross

**Affiliations:** 1 Tissue Injury and Repair Group, 6th Floor, Centre for Inflammation Research, University of Edinburgh Medical School, Teviot Place, Edinburgh EH8 9AG, UK; 2 Department of Pathology, University of Edinburgh, Royal Infirmary of Edinburgh, 51 Little France Crescent, Edinburgh EH16 4SA, UK

**Keywords:** extracellular matrix, integrins, interleukin-8, cytoskeleton, pancreatic cancer

## Abstract

Interactions between tumour cells and the microenvironment are increasingly recognised to have an influence on cancer progression. In pancreatic carcinoma, a highly desmoplastic stroma with abnormal extracellular matrix (ECM) protein and interleukin-8 (IL-8) expression is seen. To investigate whether the ECM may further contribute to abnormalities in the microenvironment by influencing IL-8 secretion, we cultured the Mia PaCa2 pancreatic carcinoma cell line on fibronectin. This resulted in a dose-dependent increase in IL-8 secretion, which was RGD dependent and accompanied by cell spreading and proliferation. The role of spreading was assessed by disruption of the cytoskeleton with cytochalasin D, resulting in a large increase in IL-8 secretion, which was reduced from 31- to 24-fold by fibronectin. This remarkable response was associated with inhibition of spreading and proliferation and represents a novel cytoskeletal function. To investigate whether it could be accounted for by the loss of integrin-mediated signalling, the expressed *α*5*β*1, *α*V*β*5 and *α*3*β*1 integrins were inhibited. *α*5*β*1 inhibition prevented spreading and proliferation but produced a much smaller rise in IL-8 secretion than cytochalasin D. *α*V*β*5 inhibition alone had only minor effects but when inhibited in combination with *α*5*β*1 completely abolished the response to fibronectin. These results reveal latent stimulatory effects of the *α*V*β*5 integrin on IL-8 secretion and suggest that integrin crosstalk may limit the induction of IL-8 secretion by fibronectin. However, the magnitude of IL-8 secretion induced by cytochalasin cannot be accounted for by integrin signalling and may reflect the influence of another signalling pathway or a novel, intrinsic cytoskeletal function.

A view of the tumour as a functional tissue interconnected with the microenvironment has recently emerged in which the host stroma participates in the induction, selection and expansion of neoplastic cells (Liotta and Kohn, 2001; Radisky *et al*, 2001; Pupa *et al*, 2002). The extracellular matrix (ECM) and cytokines are important mediators of this process whose net effects are determined by a complex series of interactions. Many cytokines bind to components of the normal ECM in an inactive form and are activated only on degradation of the ECM by a matrix protease (Streuli, 1999). These proteases are secreted mainly by stromal cells (Crawford and Matrisian, 1996) and may be induced by cytokines (Saren *et al*, 1996). Extracellular signals can also alter the expression of ECM proteins and cytokines, creating feedback loops that may contribute to tumour development. For example, the induction of tumour cell matrix protein production by cytokines has been demonstrated (Rettig *et al*, 1994). However, despite evidence in nonmalignant cells (Eierman *et al*, 1989), the induction of cytokine secretion in malignant cells by ECM components is yet to be investigated.

The ECM has been shown to influence multiple cellular functions including proliferation, apoptosis, adhesion, motility, gene expression and differentiation (Lupetti *et al*, 1996; Danen and Yamada, 2001; Brakebusch *et al*, 2002; Stupack and Cheresh, 2002). These responses are mediated by cellular receptors, of which the integrin family of heterodimeric glycoproteins is the largest and most ubiquitous. The ligand specificity of an integrin is influenced by both its *α* and *β* subunits, which contribute jointly to the ligand binding domain. This domain recognises ECM molecules via short peptide sequences, for example, the RGD sequence of fibronectin (Pytela *et al*, 1985). Upon ligation conformational changes and clustering occur, leading to the accumulation of cytoskeletal and signalling molecules at areas of plasma membrane termed focal contacts (Yamada and Miyamoto, 1995; Hynes, 2002). The integration of signals from multiple ECM molecules and cytokines is facilitated by the subsequent activation of signalling pathways and changes in cell shape (Horwitz *et al*, 1986; Otey *et al*, 1990; Maheshwari *et al*, 1999). Such signalling pathways function cooperatively in that they can often be activated by either integrins or soluble factors. For example, the focal adhesion kinase can be activated by growth factors or integrin clustering (Zachary and Rozengurt, 1992). MAPK signalling is also known to be induced by both integrins and soluble mitogens, overexpression of which leads to anchorage independence (Mansour *et al*, 1994; Schlaepfer *et al*, 1994). In contrast, integrin induced changes in cell shape often function to facilitate the effects of cytokines. In particular, signalling via the *α*5*β*1 integrin has been found to be required for soluble mitogens to induce proliferation (Miyamoto *et al*, 1995, 1996; Boudreau and Jones, 1999).

Pancreatic carcinoma is a highly malignant tumour in which an intense desmoplastic reaction and an abnormal stroma are commonly observed. In common with other tumours, alterations in the expression of cytokines and ECM proteins have been demonstrated (Riedl *et al*, 1992; Juliano and Varner, 1993; Danen *et al*, 1994; Mizejewski, 1999). In particular, marked increases in fibronectin, type I collagen and type III collagen levels are seen (Gress *et al*, 1995). Cytokine expression is also altered and, in particular, interleukin-8 (IL-8), which is not found in the normal pancreas, is produced (Di Sebastiano *et al*, 2000). A member of the CXC chemokine family, IL-8 has functions important to the progression of pancreatic carcinoma including stimulation of autocrine growth and angiogenesis (Shi *et al*, 1999; Le *et al*, 2000). Its production has been correlated with the metastatic potential of pancreatic carcinoma cells in nude mice (Bruns *et al*, 1999; Shi *et al*, 1999) and is regulated by factors altered in tumour microenvironments including nitric oxide, hypoxia, cytokines and acidosis (Shi *et al*, 2001; Xie, 2001). However, it is not yet known whether IL-8 production may be regulated by signals from the ECM. We previously screened a range of pancreatic carcinoma cell lines and found that Mia PaCa2 cells produce significant levels of IL-8 (Wigmore *et al*, 2002). Here, we describe the influence of fibronectin on IL-8 secretion and the secretion-associated phenotypic changes of cell spreading and proliferation. The relationship between cytoskeletal organisation and IL-8 secretion and the contribution of integrins to this relationship is further assessed.

## MATERIALS AND METHODS

### ECM and antibodies

The ECM molecules and control molecules employed were: type I collagen (Southern Biotech, UK), fibronectin (Sigma, UK), BSA (Sigma, UK), GRGDSP peptides and GRADSP peptides (Calbiochem-Novabiochem, Nottingham, UK). Antibodies and specificities were P1B5 (*α*3 integrin, Gibco-BRL, UK), CLB-705 (*α*5 integrin, Chemicon, UK), AMF-7 (*α*V integrin, Immunotech, UK), P1F6 (*α*V*β*5 integrin, Gibco-BRL, UK) and P4C10 (*β*1 integrin, R&D systems, UK). Additional antibodies used in flow cytometry were TS2/7 (*α*1 integrin, T Cell Sciences), AK7 (*α*2 integrin, Serotec), GoH3 (*α*6 integrin, Serotec), 23C6 (*α*V*β*3 integrin, kind gift from M Horton, cf. Horton *et al*, 1985), TS2/16 (*β*1 integrin, kind gift from J Sanchez-Madrid, cf. Arroyo *et al*, 1992), CD61 (*β*3 integrin, Dako), 3E1 (*β*4 integrin, Chemicon) and mouse Ig isotype controls (Sigma, UK). Cytochalasin D was obtained from Sigma, UK.

### Cell culture

The Mia PaCa2 human pancreatic carcinoma cell line was obtained from the European Tissue Culture Collection (ECACC, Porton Down, UK). Cells were maintained in 250 cm^2^ flasks (Costar, UK) with Dulbecco's modified Eagle's medium (Gibco-BRL, UK) plus 5% FCS. Culture media were supplemented with 50 IU ml^−1^ penicillin, 50 *μ*g ml^−1^ streptomycin and 2 mM L-glutamine (Gibco-BRL, UK). Cell culture flasks were incubated at 37°C in a 5% CO_2_ in air atmosphere and the medium changed every 3–4 days.

Before use in experiments, cell viability and concentration was assessed by trypan blue exclusion. Cells were diluted to a concentration of 1 × 10^5^ cells ml^−1^ in serum-free medium containing soluble molecules where appropriate. A total of 1.2 × 10^5^ cells were then added to each experimental well in a 24-well plate (Costar, UK). Viability was greater than 98% in all experiments. Cells were incubated for 72 h. Multiwell plates were then centrifuged at 300 **g** for 5 min. The resulting supernatants were removed and centrifuged at 13 000 r.p.m. for 1 min in preparation for ELISAs.

For experiments with coated plates, flat-bottomed 24-well plates (Nunclon) were coated with 250 *μ*l well^−1^ of a range of concentrations of the above ECM proteins diluted in PBS. Plates were then dried overnight at room temperature in a laminar flow air cabinet. Nonspecific binding sites on well surfaces were blocked by the application of 10% BSA. Wells coated with BSA alone served as negative controls. Following incubation for 1 h at 37°C, BSA was discarded and wells washed twice with PBS prior to use. Cell shape was examined at 0, 24, 48 and 72 h of culture using an inverted light microscope.

### IL-8 ELISAs

Interleukin-8 ELISAs were performed as described previously (Wigmore *et al*, 1997). Plates were read at a wavelength of 450 nm using the Dynatech MR5000 plate reader and Assayzap interpretation program. Samples and standards were tested in triplicate and the values given represent the mean values for each sample as calculated from the standard curve.

### Cell counts

Cell counts were carried out by a direct count of cells within marked fields in the inverted light microscope. Plates were examined for comparable fields, which were then marked with a fine-tipped laboratory marker and the magnification was increased to × 20. A graticule was attached to the microscope and lined up with an identifiable feature of the dot and cells within the grid of the graticule counted. Cells were initially counted 4 h after seeding to allow for settling to the bottom of the well, and again after 72 h under experimental conditions. To allow for intrawell variation, four fields per well with 80–120 cells per field were counted. On replacing the field and recounting errors were less than 5%.

### Flow cytometry

Following removal from culture flasks cells were resuspended in PBS+1% BSA+azide and counted before being processed for flow cytometry using a panel of antibodies. The secondary antibody used was sheep anti-mouse fluoroisothiocyanate conjugate (FITC, Sigma, UK). Samples were analysed using a Coulter Epics XL-MCL flow cytometer.

### Statistical analysis

Results were analysed with a two-tailed Mann–Whitney test. *P*-values of <0.05 were taken to indicate statistical significance.

## RESULTS

### Effects of fibronectin

**Figure 1:**
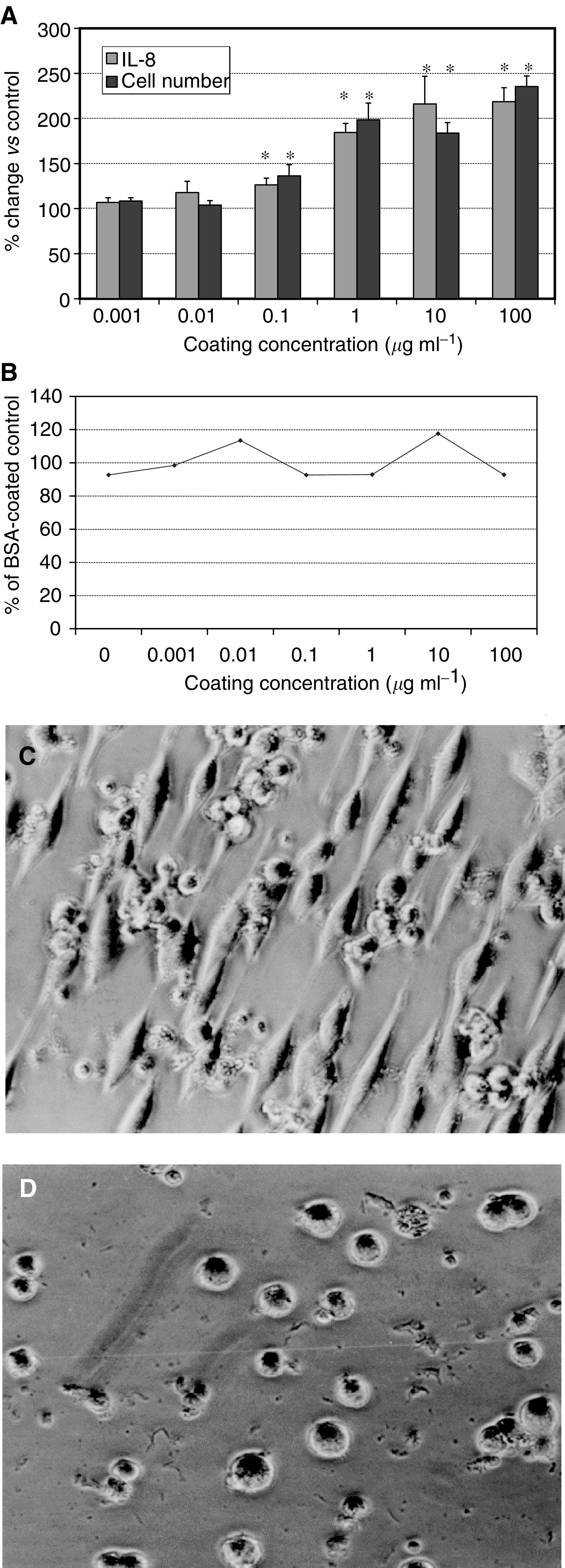
Effect of fibronectin and type I collagen on Mia PaCa2 cell IL-8 secretion, number and spreading. Culture of Mia PaCa2 cells on fibronectin for 72 h produced a dose-dependent increase in IL-8 and cell number (**A**). However, when expressed as IL-8 per cell, this was not significant (**B**). Values are expressed as a percentage of those obtained for culture on BSA. When cultured on fibronectin cells took on a fusiform, spread appearance (**C**) in comparison with the rounded appearance seen when cultured on BSA (**D**). ^*^P<0.05 *vs* untreated control.

The effect of fibronectin on IL-8 secretion by Mia cells was determined by culture in matrix-coated wells. Culture on fibronectin for 72 h resulted in a dose-dependent increase in IL-8 secretion, which was statistically significant (*P*<0.05) for coating concentrations of 0.1 *μ*g ml^−1^ and higher and plateaued at a concentration of 10 *μ*g ml^−1^ (Figure 1A and B

).

Simultaneous assay of cell numbers revealed a dose-dependent increase in numbers (Figure 1A). This was significant for coating concentrations of 0.1 *μ*g ml^−1^ and above (*P*<0.05). When IL-8 secretion was expressed as a function of cell number, fibronectin had no significant effect (Figure 1B).

When grown on fibronectin, Mia PaCa2 cells grew as single, spread cells, which were fusiform in shape (Figure 1C). In 10% BSA-coated wells, no cell spreading was observed and cells maintained a rounded morphology (Figure 1D and E)

**Figure 2:**
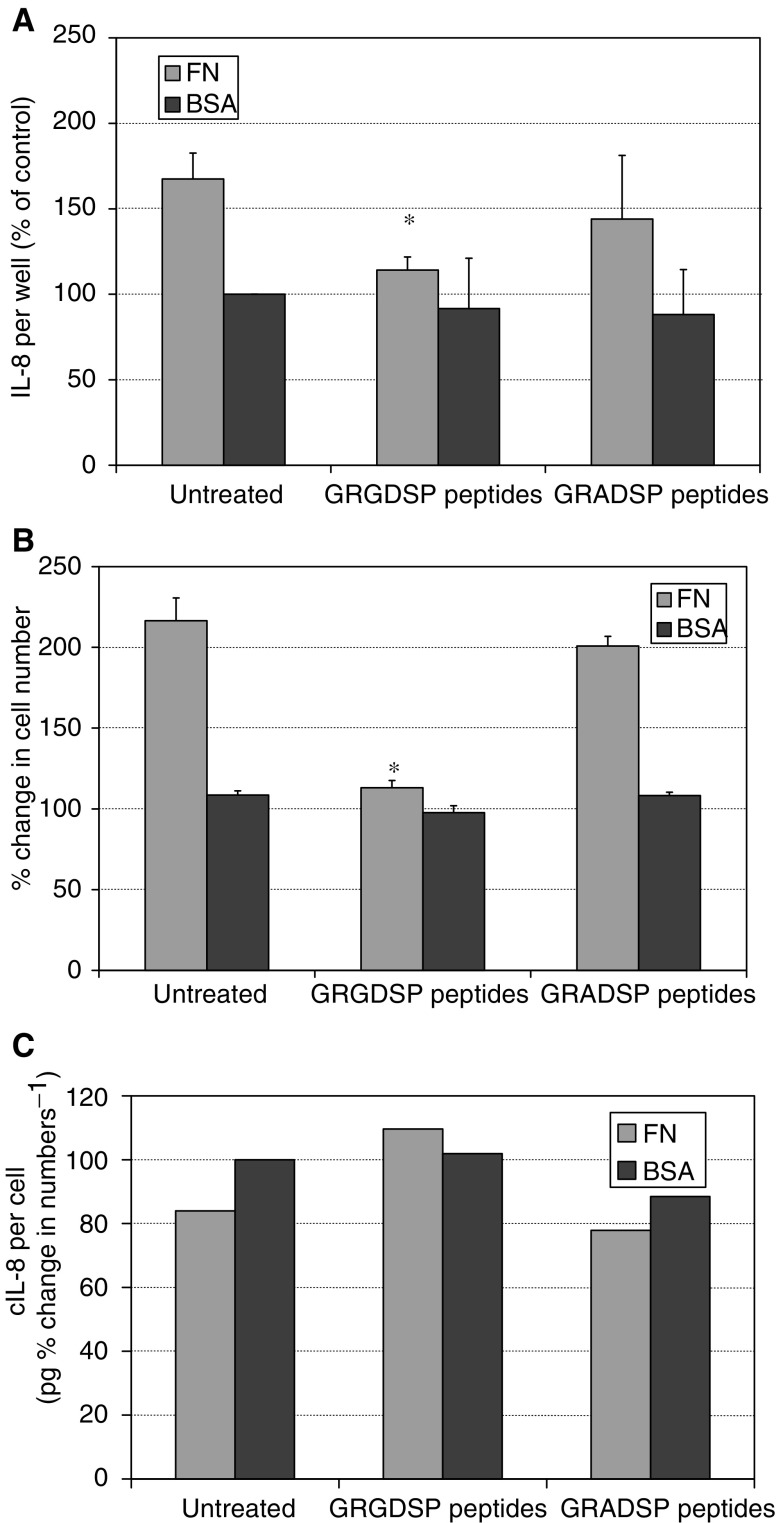
GRGDSP inhibition experiments. Following 72 h of culture GRGDSP peptides inhibited fibronectin-induced IL-8 secretion (**A**) and proliferation (**B**). No significant effect was seen with control GRADSP peptides. When IL-8 was expressed per percentage change in cell numbers, fibronectin resulted in a small drop in secretion in these experiments. This was abolished by GRGDSP peptides but not by GRADSP, suggesting that the fibronectin RGD motif is critical to its effects. ^*^P<0.05 *vs* untreated control.

Culture of Mia PaCa2 cells for 72 h in the presence of soluble GRGDSP peptides abolished the effects of fibronectin on IL-8 secretion, proliferation and spreading (Figure 2A–C

). Control GRADSP peptides did not affect the response, demonstrating that the influence of fibronectin was RGD dependent.

**Figure 3:**
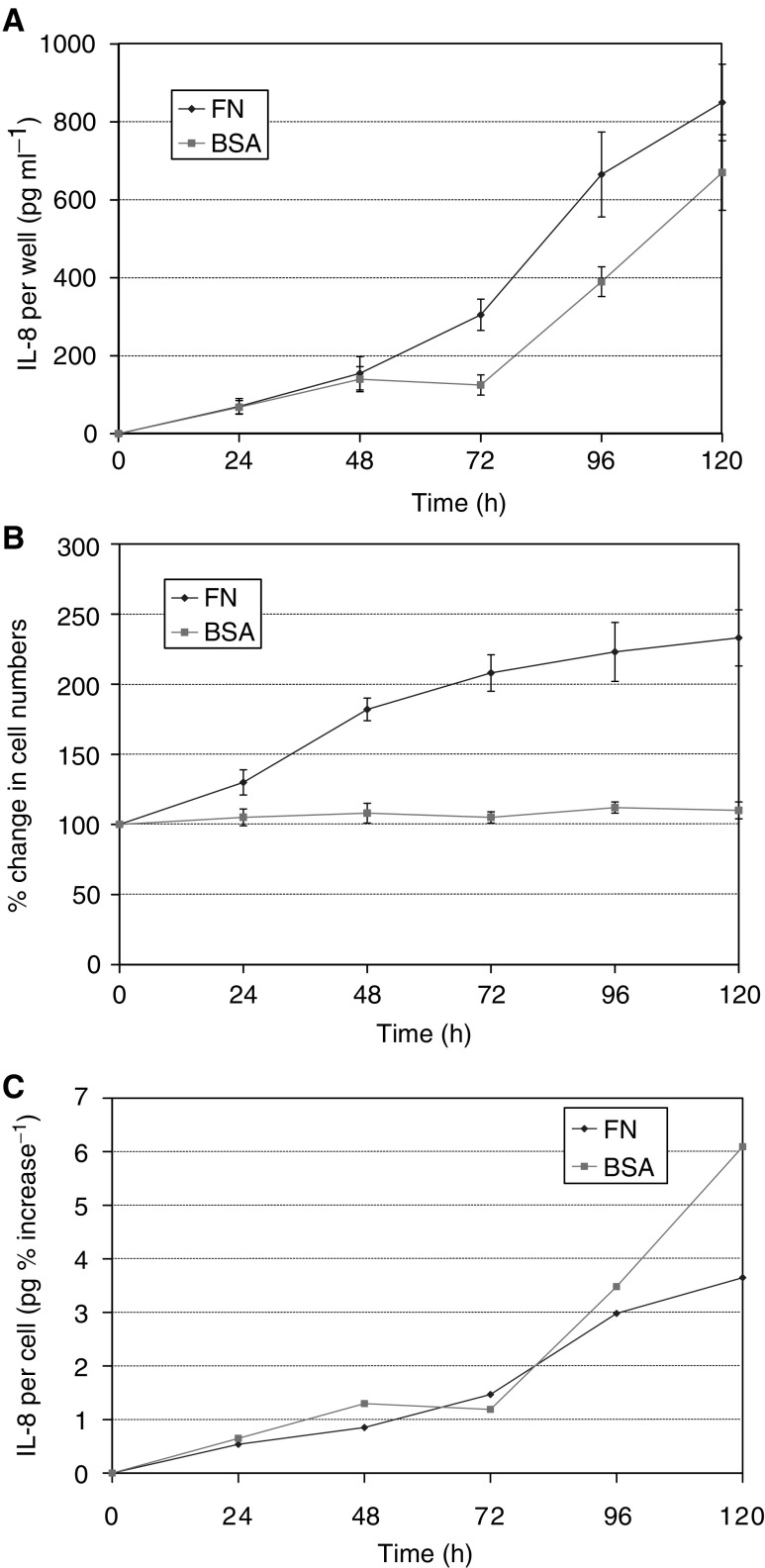
Fibronectin time course. An increase in the rate of IL-8 secretion was observed in wells coated with 1 *μ*g ml^−1^ fibronectin before any rise in secretion in BSA-coated wells (**A**). Interleukin-8 levels continued to rise at the same rate after 72 h in fibronectin- and BSA-coated wells. Cell numbers rose only in fibronectin-coated wells and slowed after 72 h (**B**). However, when results were expressed as IL-8 per cell (**C**), it was apparent that at 96 and 120 h secretion was decreased in fibronectin-coated wells.

To further investigate the relationship between cell numbers and IL-8 secretion in fibronectin-coated wells, time-course experiments were performed. From 0 to 48 h of culture, the levels of IL-8 per well increased at a constant rate and were approximately equal in fibronectin- and BSA-coated wells (Figure 3A

). From 48 to 72 h, the rate of IL-8 secretion was greater in fibronectin-coated wells. Subsequently, the rate of secretion was similar in both fibronectin- and BSA-coated wells. Cell numbers rose only in fibronectin-coated wells, with a 235% increase over 120 h (Figure 2B). When IL-8 per cell was assessed, the overall effect of fibronectin was to decrease secretion (Figure 3C). Cell spreading on fibronectin was apparent from 4 h onwards, and did not change over the course of the experiments.

These findings suggested firstly that cell proliferation was not dependent on IL-8 secretion, as cell numbers in fibronectin-coated wells rose before IL-8 levels differed from those in BSA-coated wells, and secondly that IL-8 secretion was not dependent on cell numbers, as rates of secretion per cell differed in fibronectin- and BSA-coated wells. However, it remained possible that both IL-8 levels and proliferation might be separately influenced by spreading. Actin polymerisation and cytoskeletal organisation were therefore inhibited with cytochalasin D.

### Inhibition of the cytoskeleton

**Figure 4:**
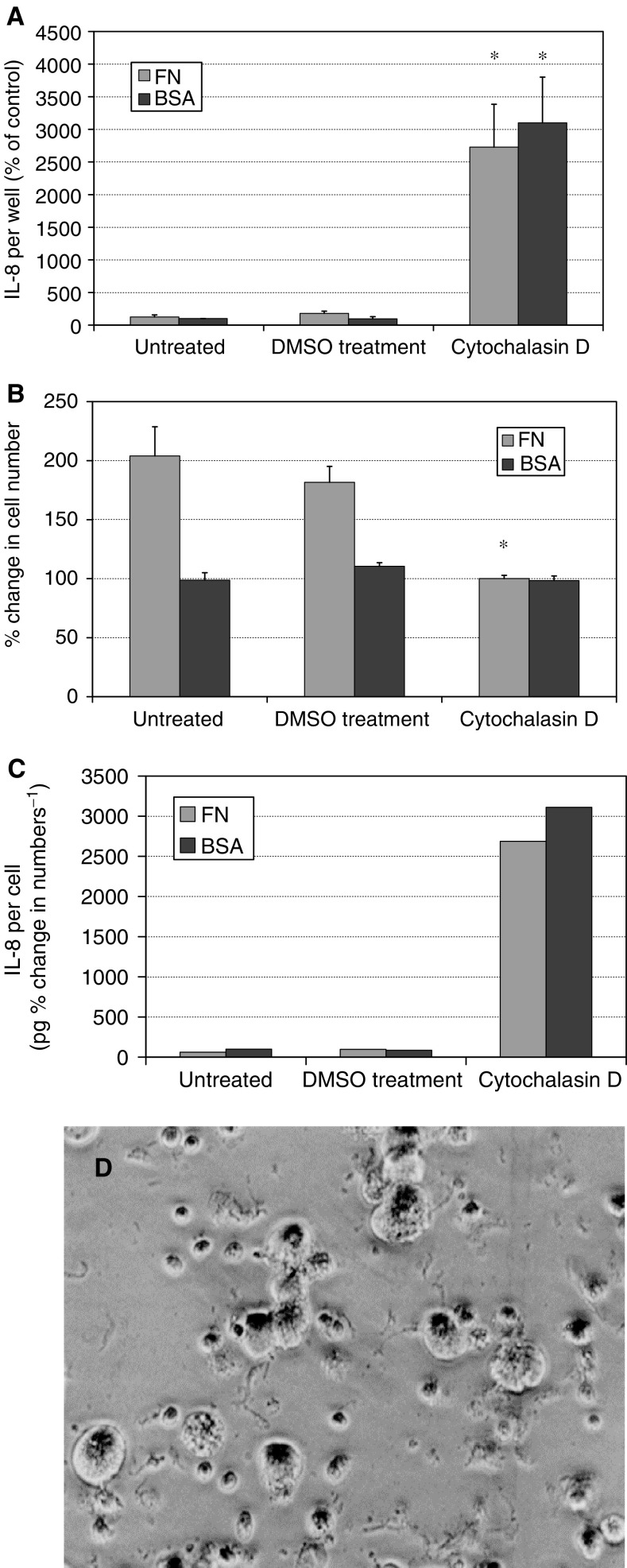
Treatment of Mia PaCa2 cells with 1 *μ*g ml^−1^ cytochalasin D. Culture of Mia PaCa2 cells for 72 h in the presence of cytochalasin D resulted in a 24-fold increase in IL-8 secretion on fibronectin-coated wells and a 31-fold increase in IL-8 secretion on BSA-coated control wells (**A**). Cytochalasin treatment also inhibited proliferation in fibronectin-coated wells in comparison with no treatment or treatment with the cytochalasin carrier vehicle DMSO (**B**). When IL-8 levels were expressed as a fraction of the percentage change in cell numbers, a large increase in secretion was still apparent and remained lower on fibronectin than on BSA. Fibronectin-induced cell spreading was prevented by cytochalasin (**D**). ^*^P<0.05 *vs* untreated control.

Cells were cultured for 72 h on fibronectin- or BSA-coated wells in the presence of 1 *μ*g ml^−1^ of cytochalasin D. This resulted in an increase in IL-8 secretion by a factor of 24-fold in fibronectin-coated wells and by a factor of 31-fold in BSA-coated wells (Figure 4A

). In wells treated with the cytochalasin D carrier vehicle DMSO, only a small increase in IL-8 secretion was observed. In contrast, addition of cytochalasin D to wells coated with fibronectin prevented rather than increased fibronectin-induced cell proliferation (Figure 4B). In BSA-coated control wells, cytochalasin D had no significant effect on cell numbers. When IL-8 was expressed as pg per percentage change in cell number, increases in IL-8 secretion of similar ratios were seen and fibronectin retained its inhibitory effect (Figure 4C). Cytochalasin did not induce loss of adherence or obvious apoptosis over the time period examined. DMSO slightly inhibited fibronectin-induced cell proliferation but had no effect in BSA-coated control wells.

Cytochalasin D prevented fibronectin-induced cell spreading (Figure 4D). On fibronectin-coated wells, the cells firmly adhered to the substratum. DMSO did not affect cell shape.

### Expression of adhesion molecules by Mia PaCa2 cells

Flow cytometry was performed to identify integrins, which might be responsible for the cytoskeleton- and fibronectin-induced effects. The *α*3 integrin was expressed by 79.2% of Mia PaCa2 cells, *β*1 integrin by 55.5%, *α*V*β*5 integrin by 27.8%, *α*V integrin by 25.6% and the *α*5 integrin by 19.7%. All other integrins were expressed by less than 5% of cells.

To further investigate the relationship between IL-8 secretion, cell proliferation and spreading, inhibition experiments were performed.

### Integrin inhibition experiments

#### *β* Integrins

**Figure 5:**
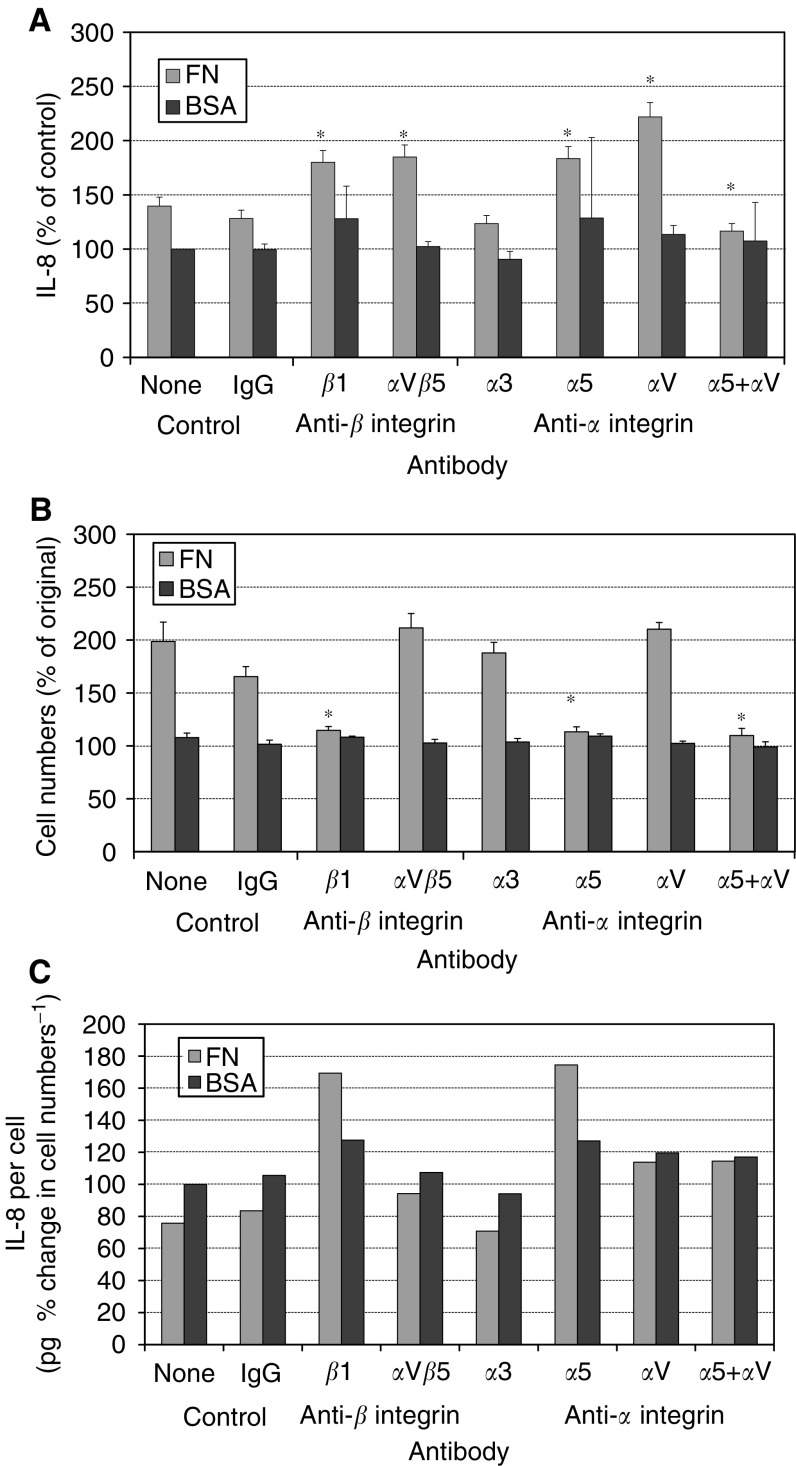
Effect of soluble anti-integrin antibodies on fibronectin-induced IL-8 secretion. Cells were cultured on either fibronectin or BSA substratums for 72 h prior to assays. The effects of fibronectin on both IL-8 secretion (**A**) and cell numbers (**B**) were abolished by both lone anti-*β*1 integrin antibodies and by anti-*α*5 and -*α*V antibodies used in combination. Use of the anti-*α*5 antibody alone did not affect IL-8 levels but abolished fibronectin-induced cell proliferation. Single use of the anti-*α*V antibody resulted in increased IL-8 levels but did not affect proliferation. When IL-8 levels were expressed as a fraction of the percentage change in cell numbers, secretion was decreased by fibronectin in these experiments (**C**). This decrease was not affected by control IgG or anti-*α*3 or -*α*V*β*5 antibodies. Anti-*β*1 and *α*5 antibodies resulted in an increase in IL-8 secreted per cell, which was greater on fibronectin than on BSA. Anti-*α*V antibodies both alone and in combination with anti-*α*5 abolished the difference between fibronectin and BSA, although in the case of anti-*α*V alone this was due to an identical increase in cell proliferation and IL-8 secretion, whereas anti-*α*V and -*α*5 in combination abolished both. ^*^P<0.05 *vs* untreated control.

Addition of either anti-*α*V*β*5 (P1F6) or anti-*β*1 (P4C10) integrin antibodies to fibronectin-coated wells resulted in an increase in IL-8 levels in comparison to untreated or IgG-treated control wells following 72 h of culture (Figure 5A

). Anti-*β*1 integrin antibodies also increased IL-8 levels in BSA-coated control wells. Nonspecific IgG antibodies did not significantly affect IL-8 secretion in either fibronectin- or BSA-coated wells.

Cell proliferation was significantly inhibited (*P*<0.05) only by anti-*β*1 integrin antibodies, despite a slight decrease seen in IgG-treated control wells (Figure 5B). In contrast with its effects on IL-8 secretion, the anti-*α*V*β*5 antibody P1F6 did not inhibit proliferation. Neither anti-*β*1 nor anti-*α*V*β*5 integrin antibodies had any effect on cell numbers in BSA-coated control wells. When results were interpreted as IL-8 per cell, fibronectin was observed to decrease IL-8 secretion. The addition of anti-*β*1 integrin antibodies reversed this effect, increasing IL-8 levels to 168% of control, while increasing levels of IL-8 produced by cells cultured on BSA to only 128% of control (Figure 5C). Anti-*α*V*β*5 integrin antibodies did not significantly affect IL-8 per cell.

Cell morphology was unaffected by *α*V*β*5 inhibition with cells remaining fully spread on fibronectin. Culture in the presence of anti-*β*1 integrin antibodies resulted in a round, unspread morphology. Neither anti-*α*V*β*5 integrin nor anti-*β*1 integrin antibodies affected cell morphology in BSA-coated wells. In summary, blockade of the *β*1 integrin resulted in an increase in IL-8 secretion on fibronectin and interaction with the *β*1 integrin was required for fibronectin-induced cell proliferation and spreading, whereas inhibition of *α*V*β*5 had little effect.

#### *α* Integrins

At 72 h, IL-8 levels in wells coated with fibronectin were increased by both anti-*α*5 (CLB-705) and anti-*α*V (AMF-7) antibodies (Figure 5A). Anti-*α*5 antibodies also increased the amount of IL-8 per well in wells coated with BSA. Anti-*α*5 and anti-*α*V antibodies in combination produced a significant inhibition of the effect of fibronectin on IL-8 secretion but did not influence IL-8 secretion in BSA-coated wells. Anti-*α*3 integrin (P1B5) and nonspecific IgG antibodies did not affect IL-8 secretion.

Cell proliferation was inhibited in wells treated with anti-*α*5 integrin antibodies alone and in combination with anti-*α*V antibodies (Figure 5B). Anti-*α*V integrin antibodies alone, anti-*α*3 and nonspecific IgG antibodies did not affect cell numbers in fibronectin-coated wells. None of the antibodies used affected cell numbers in BSA-coated wells. When IL-8 was expressed per percentage change in cell number, anti-*α*5 integrin antibodies were observed to have a similar effect to anti-*β*1 antibodies, increasing IL-8 secretion in fibronectin-coated wells more than in BSA-coated wells (Figure 5C). Anti-*α*V integrin antibodies both alone and in combination with anti-*α*5 antibodies did not affect IL-8 per cell. However, it should be noted that alone, this was due to a lack of effect on the response to fibronectin and in combination with anti-*α*5 antibodies due to a complete inhibition of the responses to both fibronectin and anti-*α*5 antibodies.

Fibronectin-induced cell spreading was inhibited by the addition of anti-*α*5 integrin antibodies but not by anti-*α*3 integrin, anti-*α*V or nonspecific IgG antibodies at either 1 or 2 *μ*g ml^−1^ concentrations.

In summary, the *α*V integrin appears to stimulate IL-8 secretion when the *α*5 integrin is inhibited and, consequently, cell spreading and proliferation are prevented. This effect is apparent on both BSA and fibronectin but greater on the latter, in contrast with the effect observed on treatment with cytochalasin, when fibronectin decreased IL-8 production.

## DISCUSSION

When Mia PaCa2 cells were cultured on fibronectin, a response that involved IL-8 production, proliferation and spreading was observed. All of these effects were RGD dependent, consistent with the known importance of the RGD motif to fibronectin-induced proliferation and spreading and its requirement for cytokine induction in certain cell types (Takizawa *et al*, 1995). Taken with the close relationship between increases in IL-8 production and cell numbers, which suggested that IL-8 secretion may directly reflect of cell number, this raised the possibility that a single, RGD-dependent mechanism might mediate the entire cellular response. However, time-course experiments revealed that the overall effect of fibronectin was to decrease IL-8 secretion per cell, as after 72 h IL-8 levels in fibronectin-coated wells did not continue to diverge from levels in BSA-coated wells despite the presence of more cells. This implies that secretion is influenced by another factor, as was the relationship purely direct IL-8 production per cell would remain constant regardless of cell number. Crosslinking of the *α*3*β*1 integrin has been demonstrated to limit IL-8 secretion in epithelial cells, and integrin ligation might therefore directly inhibit cytokine production. However, increased cell density has also been reported to limit IL-8 secretion in human polymorphonuclear leucocytes (Hattar *et al*, 2001) and could also play a role, although in pancreatic tumours a high cell density is associated with increased IL-8 secretion (Shi *et al*, 2001). It is therefore more likely that increasing cell density would stimulate Mia PaCa2 cell secretion between 48 and 72 h, shortly after the period of maximum proliferation on fibronectin. Cell cycling may also influence IL-8 production, and has been reported to inhibit IL-8 secretion in keratinocytes (Wilmer and Luster, 1995). This regulation of IL-8 production clearly differs from the normal pancreas, which does not secrete IL-8 (Di Sebastiano *et al*, 2000), potentially due to microenvironmental influence or a regulatory pathway lost in tumour cells.

The spreading observed in cells cultured on fibronectin suggested that cytoskeletal signalling might play a role in Mia PaCa2 cell IL-8 regulation. Previous studies have suggested that in functional terms, cytoskeletal signalling is mainly facilitative to other pathways (Miyamoto *et al*, 1995, 1996). We were therefore surprised to find that inhibition of the cytoskeleton with cytochalasin D resulted in such a large increase in IL-8 secretion and yet did not abolish the inhibitory effect of fibronectin. Of studies that have examined the effect of cytochalasin on cytokine production, only one has documented a similar effect, in which it was found that T-lymphocyte activation and IL-2 production were stimulated by binding of cytochalasin to high-affinity sites in actin (Geppert and Lipsky, 1990). This suggests that IL-8 induction by cytochalasin could be due to the loss of an intrinsic inhibitory effect of the cytoskeleton, rather than loss of facilitation of another inhibitory stimulus. However, in contrast to Mia PaCa2 cells, T-cell proliferation was not inhibited by cytochalasin, which may reflect the fact that proliferation in our system differs by being fibronectin, and therefore cell shape, dependent. Whatever the mechanism, it is clear that there is significant latent potential for cytoskeletal dysregulation to induce IL-8 production. That fibronectin limited this in our experiments is consistent with its proposed antitumorigenic role and suggests that it may be capable of influencing IL-8 production via a noncytoskeletal mechanism, possibly mediated via the *α*5*β*1 or *α*V*β*5 integrins (Taverna *et al*, 1998; Armstrong and Armstrong, 2000).

When either subunit of *α*5*β*1 was inhibited on either fibronectin or BSA, a similar qualitative effect to that of cytochalasin was observed, with increased secretion of IL-8 per cell. On fibronectin, spreading and proliferation were also inhibited and these similarities suggest that loss of *α*5*β*1 function could partially account for the effect of cytoskeletal inhibition. However, although the magnitude of the rise in IL-8 was significant, with secretion per cell increasing to 168% of BSA controls, it was far less than that induced by cytochalasin. This suggests that although *α*5*β*1 can stimulate cytoskeletal organisation and thereby limit IL-8 secretion, the cytoskeleton must influence secretion by at least one additional mechanism, consistent with the finding that fibronectin maintains an inhibitory effect despite cytochalasin treatment. A range of growth factors and cytokines are known to signal via the cytoskeleton and some, for example, IL-10 and TGF*β*, are known to inhibit IL-8 secretion (de Waal Malefyt *et al*, 1991; Mendez-Samperio *et al*, 2002). These cytokines have also been implicated in pancreatic carcinogenesis (Bellone *et al*, 1999; Nicolas and Hill, 2003). Interleukin-8 production secondary to the loss of their effects may therefore be an alternative explanation to the loss of intrinsic cytoskeletal function, which might account for the effect of cytochalasin. In either case, the evidence suggests that the cytoskeleton integrates several inputs in the regulation of IL-8 production.

As the only other fibronectin receptor expressed by Mia PaCa2 cells, the *α*V*β*5 integrin is highly likely to be responsible for the effects seen on *α*5*β*1 inhibition. This is supported by the abolition of all cellular responses to fibronectin by dual inhibition of *α*5*β*1 and *α*V*β*5. Therefore, *α*V*β*5 appears to have a stimulatory effect on IL-8 secretion, which is normally inhibited or masked by *α*5*β*1 function. This is a novel role for the *α*V*β*5 integrin and also for interintegrin crosstalk, which has previously been shown to influence cell adhesion and spreading, but never cytokine secretion (Marshall *et al*, 1995; Bax *et al*, 2003). In turn, it suggests that *α*5*β*1 normally functions to limit IL-8 secretion per cell, while at the same time increasing proliferation and therefore the number of secreting cells. Such a role is also unreported in other systems, where *α*5*β*1 mediates the induction of cytokines. However, a model for inhibition does exist in the *α*3*β*1 integrin, which has been shown to limit IL-8 induction by cytokines in epithelial cells (Lubin *et al*, 2003). Whether the effect of *α*5*β*1 is cytoskeleton dependent or independent is unclear. In favour of the former, it is clear that inhibition of either *α*5*β*1 or the cytoskeleton results in a large increase in IL-8 secretion and *α*5*β*1 induces cytoskeletal organisation through induction of spreading. However, even when the cytoskeleton was disrupted, fibronectin maintained an ability to limit IL-8 secretion, in contrast to the effect seen on *α*5*β*1 inhibition, when IL-8 levels in fibronectin-coated wells rose to above those in BSA-coated wells. Similarly, the crosstalk between *α*5*β*1 and *α*V*β*5 may be mediated by any of the several mechanisms. At the most basic level, inhibition of *α*5*β*1 may result in an increase in the number of available RGD sites to which *α*V*β*5 can bind. Alternatively, the integrins could interact via shared signalling pathways, alterations in ligand affinities or by altering fibronectin assembly, and thereby extracellular feedback (Tomatis *et al*, 1999; Danen *et al*, 2002; Perruzzi *et al*, 2003).

The latent functions of the cytoskeleton and integrins we have described may have significant implications, as it is increasingly recognised that the activation of latent pathways can cause failure of otherwise promising anticancer agents. The targeting of nonredundant signalling ‘nodes’ has subsequently been suggested and integrins have been proposed as one of these (Cui and Lee, 2004). However, our results suggest they are perhaps not as functionally exclusive as previously thought and that major signal integration may occur at the level of the cytoskeleton. Despite this, combination therapies aimed at integrins and other mediators of cell–microenvironment interactions have shown promising results in other conditions (Anaya-Prado *et al*, 1998). The identification of the combinations of extracellular and intracellular effects of the microenvironment that are critical to tumour progression should facilitate such approaches in cancer.
